# circRNA-02213 Regulates Milk Fat Synthesis in Bovine Mammary Epithelial Cells via ACSS2

**DOI:** 10.3390/genes16111351

**Published:** 2025-11-09

**Authors:** Meixia Sui, Gaofei Duan, Zongwei Wang, Shuhua Guo, Jingjing Fan

**Affiliations:** College of Biology and Oceanography, Weifang University, Weifang 261061, China; 18561502227@163.com (G.D.); 13905360249@126.com (Z.W.); jiujiuxue66@163.com (S.G.); fanjingjing2008@163.com (J.F.)

**Keywords:** circRNA-02213, miR-328, ACSS2, milk fat metabolism, bovine mammary epithelial cells

## Abstract

Background: In the bovine mammary gland, de novo fatty acid synthesis is a critical process for milk fat production, in which acetyl-CoA synthetase 2 (ACSS2) serves as a key enzyme by converting acetate into acetyl-CoA. This metabolic pathway is intricately regulated by non-coding RNAs, particularly through the competitive endogenous RNA (ceRNA) mechanism.Purpose: To elucidate the regulatory role and molecular mechanism of the circRNA-02213/miR-328/ACSS2 axis in the lipid metabolism of bovine mammary epithelial cells (BMECs). Methods: Bioinformatic prediction and dual-luciferase reporter assays were employed to verify the targeting interactions among circRNA-02213, miR-328, and ACSS2. In BMECs, qRT-PCR, Western blot, triglyceride/cholesterol quantification, Oil Red O staining, and cell proliferation assays were used to evaluate the effects of this axis on key lipid-metabolic indices and cellular phenotypes. Results: circRNA-02213 functioned as a molecular “sponge” that sequestered miR-328, thereby upregulating ACSS2 expression. Functionally, circRNA-02213 overexpression markedly promoted triglyceride and cholesterol synthesis, lipid droplet accumulation, and BMEC proliferation; whereas miR-328 exerted significant inhibitory effects on these lipid-metabolic processes and cell proliferation. Conclusions: This study demonstrates that circRNA-02213 acts as a ceRNA to relieve miR-328-mediated repression of ACSS2, constituting a critical network that regulates milk fat synthesis and metabolism. The circRNA-02213/miR-328/ACSS2 axis represents a potential molecular target for improving milk lipid quality in ruminants.

## 1. Introduction

The significant content of chain fatty acids makes milk highly nutritious. In the mammary gland tissue of ruminants, short- and medium-chain fatty acids are produced primarily through the de novo synthesis of fatty acids [1,2]. This process uses acetyl-CoA as a substrate and is catalyzed by acetyl-CoA carboxylase (ACACA) and fatty acid synthase (FASN) to form C16:0 saturated palmitic acids [3]. Among them, acetyl-CoA is predominantly converted into acetate through being catalyzed by acetyl-CoA short-chain fatty acid synthase (ACSS). The ACSS family comprises two subtypes: ACSS1 and ACSS2. ACSS1, primarily found in the mitochondria of cells, mainly catalyzes the oxidative decomposition of acetate to provide energy. ACSS2, on the other hand, spotted in the cytoplasm and nucleus and serves as the primary subtype that catalyzes the conversion of free acetate salts to acetyl-CoA [4]. Meanwhile, the primary location for the de novo synthesis of mammalian fatty acids is in the cytoplasm [5]. Research has shown that cancer cell lines show a significantly higher expression of the ACSS2 gene in their cytoplasm compared to normal cells, while the expression of ACSS1 in mitochondria remains unchanged. Compared with normal cells, cancer cells exhibit an elevated uptake of carbon-14 labeled acetate, with ACSS2 being the prime gene responsible for promoting the conversion of carbon-14 labeled acetate into acetyl-CoA. In cancer cells, the assimilated acetate is mainly used for fatty acid synthesis, being neither metabolized into carbon dioxide through the tricarboxylic acid cycle nor used for amino acid synthesis. Catalyzed by ACSSs, acetate combines with coenzyme A (CoA) to form acetyl-CoA, which then participates in the synthesis of fatty acids and the acetylation of histone. Lipid synthesis is a crucial process for maintaining cell survival and proliferation, and the significance of acetate in this process has garnered considerable attention. This study explores the regulatory mechanism of de novo fatty acid synthesis in the mammary gland of cows, with a particular focus on ACSS2 as the starting point. Our goal is to clarify its regulatory pathways and modes of action, thereby providing a theoretical basis for further investigations into the mechanisms involved.

Circular RNAs (circRNAs), belonging to the non-coding RNA (ncRNA) group, have recently emerged as a hot topic [6]. Since their discovery via electron microscopy in 1979, they have typically been regarded as byproducts of RNA splicing, receiving little attention due to their scarcity and the absence of functional research [7]. However, the latest technologies, such as RNA sequencing, qRT-PCR, and computational analysis, make it possible to reveal their widespread presence and tissue-specific expression due to their long half-lives. CircRNAs, therefore, have become vital post-transcriptional gene regulators by binding to microRNAs and relieving their inhibition of mRNA targets [8]. Furthermore, it is worth noting that circRNAs are capable of affecting gene expression during transcription. CircRNAs’ biological mechanism is not fully understood, yet it is commonly believed that most circRNAs are formed through a splicing mechanism known as head-to-tail dorsal splicing [9]. CircRNAs have been identified in recent studies as significant regulatory factors in various life domains, such as the biological processes of development, differentiation, and disease [10]. CircRNAs are capable of forming RNA–RNA complexes with non-coding RNAs and mRNAs, altering the function or stability of these RNA molecules, whereas circRNA–mRNA complexes have the ability to modify the stability or translation of these mRNAs [11].

Given the significance of circRNAs in the function of cow mammary glands, TargetScan and RNA hybrid software were employed to identify potential miRNA binding sites, such as miR-328. Ultimately, circRNA-02213 was identified as the research subject. Our findings indicated that circRNA-02213 functions in BMECs and acts as a competitive adsorbent for miR-328, functioning as ceRNA. Therefore, this study aimed to determine whether circRNA-02213 directly interacts with miR-328, to investigate how this interaction affects the stability and expression of the ACSS2 mRNA, and to clarify the biological role of circRNA-02213 in regulating lipid metabolism and milk fat synthesis in BMECs. Elucidating the circRNA-02213/miR-328/ACSS2 regulatory axis will provide new insights into the molecular mechanisms underlying milk fat metabolism in dairy cows.

## 2. Materials and Methods

### 2.1. Culture of BMECs

The BMECs used in this study were provided by the Ruminant Animal Laboratory of the College of Animal Science and Technology, Yangzhou University. The primary cells were received at passage 4. BMECs were cultured in DMEM/F12 medium (Thermo Fisher Scientific, Waltham, MA, USA) supplemented with 10% fetal bovine serum (FBS, Thermo Fisher Scientific, Waltham, MA, USA), 5% biological industries, 10 μg/mL hydrocortisone, 10 ng/mL of epidermal growth factor, and 10 kU/L of dual antibody (Hyclone) at 37 °C and 5% CO_2_.

The epithelial origin of the cells was confirmed by their characteristic cobblestone morphology. Furthermore, the cells were confirmed to be free of mycoplasma contamination throughout the study.

For subculturing, when cells reached approximately 90% confluence, the culture medium was aspirated. The cell monolayer was gently rinsed twice with phosphate-buffered saline (PBS). Subsequently, cells were treated with 0.25% trypsin–EDTA solution and incubated at 37 °C for 2–3 min to facilitate detachment. The trypsinization process was carefully monitored and terminated by adding complete culture medium containing 10% FBS once the majority of cells had detached. The cell suspension was then collected and centrifuged. After centrifugation, the supernatant was removed, and the cell pellet was resuspended in fresh complete medium. Finally, cells were seeded into new culture vessels at appropriate densities for subsequent experiments.

### 2.2. Determination of Triglycerides and Cholesterol

The cells for intracellular triglyceride and cholesterol detection were inoculated in a 60 mm culture dish and cultured for 48 h. After discarding the culture medium, cells were rinsed twice with pre-cooled PBS. To accurately assess lipid metabolism, intracellular triglycerides and total cholesterol were extracted and quantified using specific commercial assay kits (Triglyceride Reagent, Thermo Fisher Scientific, Waltham, MA, USA; cholesterol detection, Thermo Fisher Scientific) following the manufacturer’s protocol. The obtained values were normalized to the total cellular protein content determined by a BCA assay, ensuring comparisons were based on equivalent biomass. Untransfected BMECs cultured under identical conditions served as the control group for all experiments.

### 2.3. Oil Red O Staining

After transfection, cells were harvested from 12-well plates and stained with oil red O staining reagent (Thermo Fisher Scientific, Waltham, MA, USA). Cells underwent three washes with pre-cooled PBS, followed by adding pre-cooled 10% paraformaldehyde (*m*/*v*, 1 g of paraformaldehyde dissolved in 10 mL of ultrapure water and stirred overnight at 80 °C until completely dissolved before being stored at 4 °C for later use). After removing paraformaldehyde, we aspirated the formaldehyde and added 1 mL of 0.5% oil red O dye solution (0.05 g oil red dry powder dissolved in 10 mL of 70% ethanol solution). The solution was shaken and dissolved in a shaker at 37 °C for 6 h. After being completely dissolved, 0.2% oil red O dye solution was added. After being filtered with an M filter, the resulting solution was stored in the dark at 4 °C and then underwent a staining process at room temperature for 30 min. The next step is to discard the oil red O staining solution and wash the cells three times with PBS before taking photos for observation.

### 2.4. EdU Cell Proliferation Detection

In the logarithmic growth phase, cells exhibiting a favorable growth status were inoculated into a 6-well plate at a density of approximately 1 × 10^8^ cells per well. Once the cell confluence reached 60%, transfection was performed using pcDNA-MDNCR (the experimental group). Untransfected cells were processed in parallel and served as the control group, followed by a 24 h incubation. The study used EdU (Thermo Fisher Scientific, Waltham, MA, USA) labeling to remove the culture medium, followed by cell fixation by adding 100 μL of 4% paraformaldehyde solution to each well and incubating at room temperature for 30 min. Following the removal of the solution, cells were treated with 100% μL 2 mg/mL glycine on a shaker for 5 min. This was followed by washing cells with PBS. Subsequently, image analysis was conducted after staining and decolorization, and fluorescence microscopy was used to observe and analyze the results. The experiment was independently repeated three times to ensure statistical reliability.

### 2.5. Dual Luciferase Report Research

To determine the potential direct targeting of these sites by miR-328, an ACSS2 3′UTR fragment containing the predicted miR-328 target site was synthesized. This fragment was then inserted into the psiCHECK-2 vector (Promega, Madison, WI, USA) at a site between the Xho I and Not I sites, which is adjacent to the downstream of the luciferase gene in the sea kidney. The integrity of all constructs was verified through sequencing. Subsequently, digested 293A cells were inoculated onto a 48-well plate. At a 75% adhesion concentration of 293A cells, the PEI transfection agent was employed to introduce them into the reporter gene plasmid vector. Then, the mimic or inhibitor was transfected into the cells. Activity was assayed 48 h after transfection. PCDNA-miR-103 was co-transfected with recombinant psiCHECK-2-circ003429-W/psiCHECK-2-circ003429-Mut (pCK circ003429-W/pCK circ003429-Mut) vector into HEK293T cells, and the dual luciferase gene reporter vector assay validated the targeting relationship (Table S1). All experiments included six technical replicates for statistical reliability.

### 2.6. Quantitative Real-Time PCR

cDNA synthesis was performed using the TaKaRa PrimeScript Reverse Transcription Kit (TaKaRa, Dalian, China) with a universal reverse transcription system consisting of 500 ng of total RNA, 2 μL of 5 × Mix, 0.5 μL of Random6 primer, and 10 μL of ddH2O. The qRT-PCR program involved a 30 s incubation at 95 °C, followed by 39 cycles of a 5 s incubation at 95 °C and a 30 s incubation at 60 °C, and finally, a 10 s incubation at 95 °C and a 5 s incubation at 65 °C. The qRT-PCR was performed using SupRealQ Purple Universal SYBR qPCR Master Mix (U+) (Vazyme, Q412-02). The 2^−ΔΔCT^ method was used to calculate relative gene expression, using UXT as the internal reference (Table S2).

### 2.7. Western Blot Detection

Cellular proteins were extracted and separated with SDS-PAGE to measure protein expression levels. They were transferred onto a nitrocellulose membrane (Millipore, Billerica, MA, USA) and probed with primary monoclonal rabbit ACSS2 (Abcam, EPR8500, Cambridge, UK) or monoclonal mouse anti β-Antibodies against actin (Protein Group, 66009-1-IG, Wuhan, China). Polyclonal Goat Anti-Rabbit IgG H&L (HRP) (Abcam, ab672, Cambridge, UK) was employed as the secondary antibody. All antibodies were used following the guidance provided by the manufacturer. Finally, a chemiluminescence ECL protein imprinting system (Pierce, Appleton, WI, USA) was utilized to detect signals.

### 2.8. Immunohistochemistry

Slices were baked at 60 °C for 30 min. To remove the wax, slices were immersed in xylene for 10 min two times. Then, they were soaked in ethanol solutions of the following concentrations for 5–10 min at each stage: 100%, 95%, 85%, and 75%. For hot repair, the antigen underwent a 5-minute soak in distilled water. Specifically, the slices were soaked in a citrate buffer at pH 6.0 and microwaved at high power for 8 min. Next, the slices were cooled to room temperature and underwent three 3-min washes with PBS (pH 7.2~7.6). To inactivate endogenous enzymes, 3% H_2_O_2_ was applied and left to stand at room temperature for 10 min. Then, PBS was used again to wash the slides 3 times for 3 min each. For serum blocking, the slices were incubated in 5% sheep serum in a wet box at room temperature for 20 min. The slices were then dried without any washing. For incubation with the primary antibody, the appropriately diluted primary antibody was added dropwise. An equivalent volume of PBS was administered to the control group. Slides were refrigerated overnight at 4 °C. For incubation with secondary antibodies, slides were taken from the refrigerator and reheated at 37 °C for 1.5 h. They were rinsed 3 times for 5 min each with PBS. Following this, slides were treated with 50–100 μL of secondary antibody and incubated at 37 °C for 20 min. Regarding re-dyeing, the colored film was washed with distilled water, followed by 1–3 min of soaking in hematoxylin. After re-dyeing, it was rinsed with distilled water and PBS, resulting in a blue color. For dehydration, the film was dehydrated using alcohol at various levels (60–100%) for 5 min per level. To achieve transparency, the film was placed in xylene for 10 min twice. Subsequently, neutral gum sealing was applied for microscopic observation.

### 2.9. Data Analysis

The analysis of real-time fluorescence quantitative PCR results was conducted using the 2^−ΔΔCt^ model. The SPSS 19.0 statistics software package was used to perform statistical analyses, with data being presented as the means ± SD (standard deviation) of three independent experiments. Differences were considered significant when *p* < 0.05 and extremely significant when *p* < 0.01.

## 3. Results

**Figure 1:**
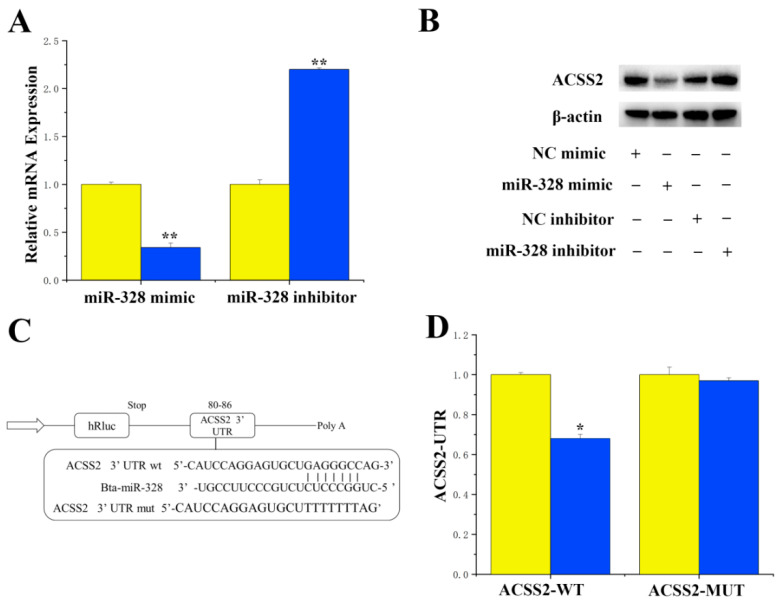
The specific targeted regulation of ACSS2 by miR-328. (**A**) The effects of mimics and inhibition of miR-328 on the expression level of INSIG1 mRNA. Expression was quantified by RT-qPCR (n = 6). (**B**) The effects of overexpression and inhibition of miR-328 on the level of ACSS2 protein. (**C**) Analysis of binding sites of miR-328 on ACSS2 3′UTR. (**D**) The effects of overexpression of miR-328 on the activity of wild-type and mutant luciferase reporter vectors of the ACSS2 gene 3′UTR. WT: a luciferase reporter vector containing wild-type ACSS2 3′UTR (80 bp to 86 bp); MUT: a luciferase reporter vector containing a mutated miR-328 binding site on ACSS2 3′UTR (n = 6). Values are presented as means ± standard errors. * *p* < 0.05; ** *p* < 0.01.

For the specific targeted regulation of ACSS2 by miR-328, miRNAs targeting ACSS2 were identified using the online software TargetScan 6.2 and the miRNA function analysis software DAVID. Our results revealed a perfect match between the 3′UTR of ACSS2 and miR-328, suggesting a potential specific targeting of ACSS2 by miR-328. Experimental findings showed that treatment with miR-328 mimics resulted in the downregulation of mRNA expression (*p* < 0.01) and the protein levels of ACSS2 in BMECs (Figure 1A,B). In addition, the 3′UTR of ACSS2 exhibited a perfectly matched binding site with miR-328 (Figure 1C). The luciferase report indicated that the overexpression of miR-328 significantly suppressed the activity of CD36 (*p* < 0.01), while the activity of ACSS2 remained unchanged in the mutant (Figure 1D).

**Figure 2:**
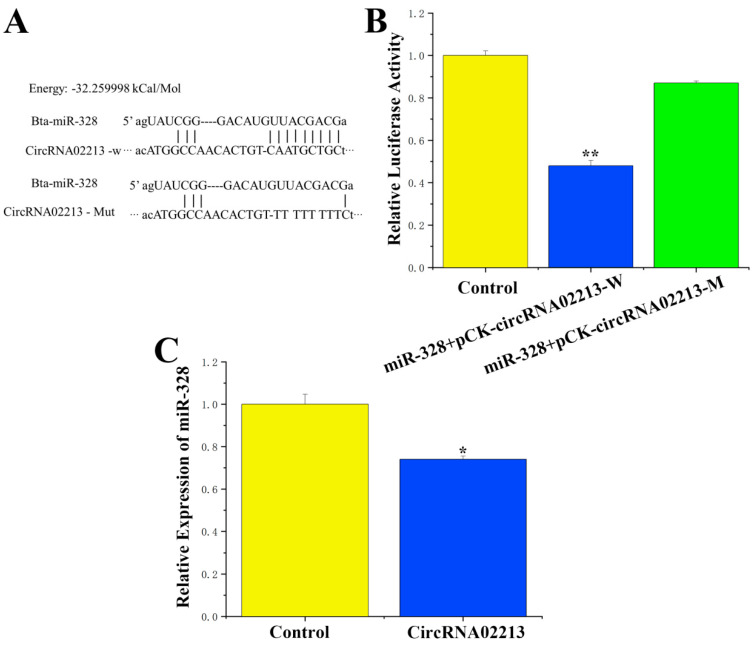
circRNA-02213 competitively binding to miR-328. (**A**) A potential miR-328 binding site in circRNA-02213. (**B**) MiR-328 mimic reduced the luciferase activity of pCK circRNA-02213 (n = 6). (**C**) CircRNA-02213 reduced the expression of miR-328 levels. Expression was quantified by RT-qPCR (n = 6). Values are presented as means ± standard errors. * *p* < 0.05; ** *p* < 0.01.

For circRNA-02213, which competitively binds to miR-328, sequence analysis revealed that circRNA-02213 originated from chromosome 13 from 10758369 to 10766830 (Tables S3 and S4), and a binding site for miR-328 was identified in this sequence. To verify the binding relationship between circRNA-02213 and miR-328, PCR was employed to amplify and recombine sequences with miR-328 binding sites into the psiCHECK-2 vector, forming a wild-type recombinant vector. Additionally, the binding sites were mutated into a mutated psiCHECK-2 vector through overlapping PCR (Figure 2A). A targeted binding relationship between circRNA-02213 and miR-328 was demonstrated using a dual luciferase reporter assay. Our findings demonstrated that miR-328 activity notably declined after co-transfection with wild-type vectors (*p* < 0.01), while no significant differences in activity were observed between the mutant vector and the control group (Figure 2B). Moreover, circRNA-02213 significantly inhibited the expression of miR-328 (*p* < 0.05) (Figure 2C). These results indicate that circRNA-02213 competitively binds to miR-328.

**Figure 3:**
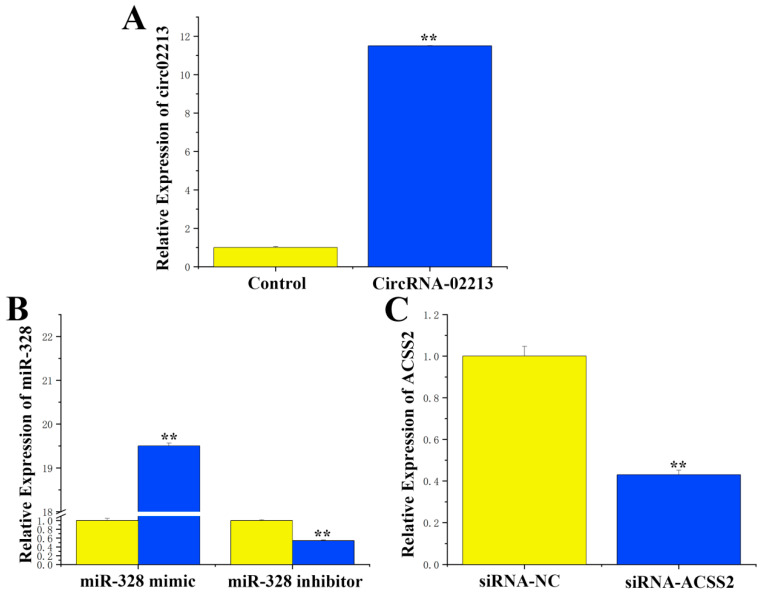
Transfection efficiency of circRNA-02213, miR-328, and siRNA-ACSS2: (**A**) CircRNA-02213 expression level after transfection with pcDNA–circRNA02213. (**B**) Detection of miR-328 after transfection with miR-328 mimic/inhibitor. (**C**) Detection of ACSS2 after transfection with siRNA-ACSS2. Expression was quantified by RT-qPCR (n = 6). Values are presented as means ± standard errors.** *p* < 0.01.

As for the transfection efficiency of circRNA-02213, miR-328, and siRNA-ACSS2, to further investigate the function of circRNA-02213 in BMECs, we successfully constructed a circRNA-02213 sequence overexpression vector (pcDNA–circRNA02213) and validated its expression efficiency. The results revealed that the expression of circRNA-02213 exhibited an 11-fold increase following transfection into cells (Figure 3A). After treatment, the expression of miR-328 mimic was upregulated by approximately 19.5 times compared to the control group. Conversely, the expression of miR-328 was downregulated by about 45% following treatment with an miR-328 inhibitor (Figure 3B). Additionally, transfecting cells with SiRNA-ACSS2 resulted in a substantial 60% decline in ACSS2 (Figure 3C). These findings suggest that the transfection efficiency of circRNA-02213, miR-328, and siRNA-ACSS2 can all be used in subsequent studies.

**Figure 4:**
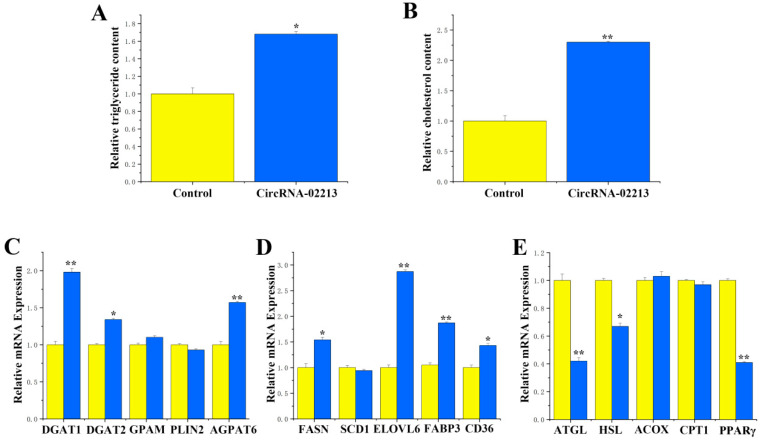
Functional validation of circRNA-02213 in BMECs: (**A**) The effect of circRNA-02213 on the level of triglyceride synthesis. (**B**) The effect of circRNA-02213 on cholesterol levels. (**C**–**E**) The effect of circRNA-02213 on the expression level of genes related to milk fat metabolism. Expression was quantified by RT-qPCR (n = 6). Values are presented as means ± standard errors. * *p* < 0.05; ** *p* < 0.01.

**Figure 5:**
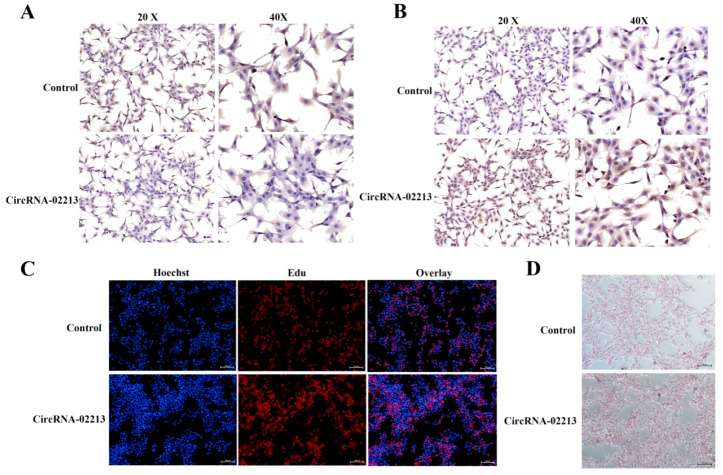
Differentiation of circRNA-02213 in BMECs: (**A**) DGAT1 protein immunohistochemistry of BMECs transfected with the circRNA-02213. (**B**) ELOVL6 protein immunohistochemistry of BMECs transfected with the circRNA-02213. (**C**) Cell differentiation dealt with circRNA-02213. (**D**) Lipid droplet accumulation was dealt with by circRNA-02213.

As for the functional validation of circRNA-02213 in BMECs, as milk fat primarily consists of triglyceride and cholesterol, we detected the secretion of triglycerides, cholesterol, and lipid droplets upon the overexpression of circRNA-02213 and found considerably higher expression of triglycerides and cholesterol (*p* < 0.01) (Figure 4A,B). Additionally, there were notable upregulations (*p* < 0.01) in the expression of genes associated with milk fat metabolism, including DGAT1, DGAT2, and AGPAT6, related to triglyceride synthesis (Figure 4C). There were also considerable rises in the expression of FASN, ELOVL6, FABP3, and CD36 genes that were associated with fatty acid synthesis (Figure 4D). Conversely, the expression of ATGL, HSL, and PPARγ associated with fatty acid oxidation was observed to be significantly downregulated (Figure 4E). In circRNA-02213-transfected cells, however, the expression of dark brown-stained DGAT1 and ELOVL6 proteins was significantly higher (Figure 5A,B). The EdU results revealed that the overexpression of circRNA-02213 resulted in an increased number of cells (Figure 5C). The oil red O staining results demonstrated a significant increase in lipid droplet accumulation under the overexpression of circRNA-02213 in BMECs (Figure 5D).

**Figure 6:**
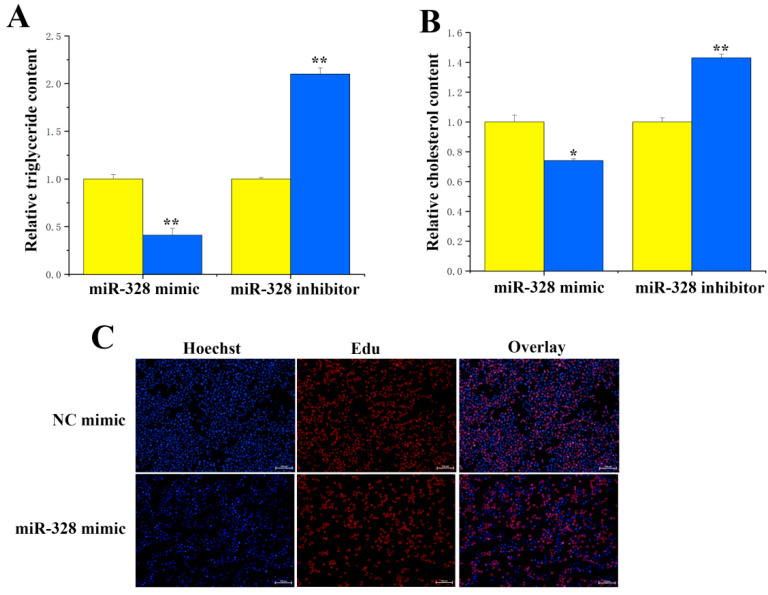
Functional validation of miR-328 in BMECs: (**A**) The level of triglyceride synthesis upon treatment with miR-328 (n = 6). (**B**) The level of cholesterol upon treatment with miR-328 (n = 6). (**C**) The accumulation of lipid droplets upon treatment with miR-328 mimic. Values are presented as means ± standard errors. * *p* < 0.05; ** *p* < 0.01.

**Figure 7:**
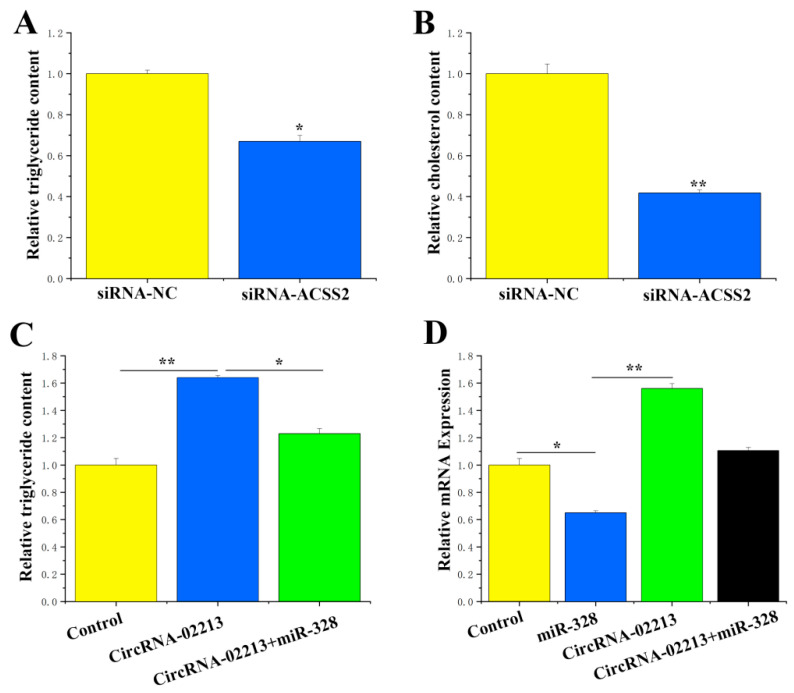
Functional validation of ACSS2 in BMECs (**A**) The level of triglyceride synthesis upon treatment with siRNA-ACSS2 (n = 6). (**B**) The level of cholesterol upon treatment with siRNA-ACSS2 (n = 6). (**C**) Triglyceride levels upon transfection with control, circRNA-11228, or circRNA-11228 + miR-103. TAG levels are compared with those of the control (n = 6). (**D**) INSIG1 expression levels upon transfection with control, miR-103, circ007071, and circRNA-11228 + miR-103 (n = 6). Values are presented as means ± standard errors. * *p* < 0.05; ** *p* < 0.01.

For the functional study of miR-328 and ACSS2 in BMECs, here, we evaluated how the overexpression and inhibition of miR-328 affected levels of triglycerides, cholesterol, and cell differentiation in BMECs. Our results indicated that suppressing miR-328 expression significantly (*p* < 0.01) lessened the difference in triglyceride content (Figure 6A). Additionally, miR-328 significantly downregulated the cholesterol concentration (Figure 6B), with inhibition of miR-328 resulting in a reduction in cholesterol content of approximately 0.7 times. The EdU results revealed that increasing miR-328 led to a lower number of cells (Figure 6C). Compared with the control group, significant reductions in the concentrations of triglycerides (*p* < 0.05) and cholesterol (*p* < 0.01) were observed in BMECs treated with ACSS2 siRNA (Figure 7A,B). It can be inferred from these findings that ACSS2 plays a role in regulating milk fat metabolism.

CircRNA-02213 regulates triglyceride levels through the adsorption of miR-328: Former studies have validated that circRNA-02213 promotes the synthesis of triglycerides in BMECs, while miR-328 inhibits it. As part of our “remedial” experiments, we attempted to demonstrate the functional regulatory relationship between circRNA-02213 and miR-328. It is evident that miR-328 could alleviate the effect of circRNA-02213-induced increases in triglycerides in cells (*p* < 0.05) (Figure 7C). In addition, miR-328 specifically targeted ACSS2. Is it possible that circRNA-02213 releases its effect on ACSS2 by adsorbing miR-328? Our findings revealed that circRNA-02213 promoted the expression of ACSS2, but miR-328 could alleviate circRNA-02213’s effect on ACSS2 (Figure 7D). Taken together, circRNA-02213 is capable of binding to miR-328 in a competitive manner, thereby alleviating the inhibitory effect of miR-328 on ACSS2 and regulating triglyceride levels in BMECs.

## 4. Discussion

In ruminant milk, half of the fatty acids are synthesized de novo, using acetic acid from carbohydrate fermentation in the rumen as the primary carbon source [12], while in non-ruminant milk, glucose serves as the main carbon source [13]. ACSS2 in the cytoplasm is responsible for catalyzing the production of acetyl-CoA from acetate and CoA, providing a substrate directly for this synthesis [14]. In mammals, acetyl-CoA serves as a crucial intermediary in the body’s carbon metabolism, exerting significant influence on energy metabolism, cellular proliferation, and the regulation of gene expression [15,16]. As a pivotal carbon provider, acetyl-CoA assumes a vital role in synthesizing fatty acids and cholesterol. The primary sources of acetyl-CoA are twofold: under normal circumstances, ATP citrate lyase (ACLY) assists in converting citric acid (derived from glucose) into acetyl-CoA, while in the absence of oxygen and nutrients, ACSS2 catalyzes the formation of acetyl-CoA from acetic acid, thereby consuming ATP [13,17]. This underscores the crucial role of ACSS2-mediated acetyl-CoA generation in organisms. During lactation, breast tissue shows significantly higher expression of ACLY and ACSS2 genes than in the dry milk stage, and this is mirrored in cancer cells as opposed to normal cells [18,19,20].

Recent research has found that cancer cells require a substantial quantity of lipids to form cell membranes. Concurrently, their rapid proliferation and growth lead to intracellular hypoxia and nutrient deficiency [21,22]. ACSS2 facilitates the formation of acetyl-CoA from acetate, providing the primary acetyl-CoA source necessary for synthesizing de novo fatty acids in cancer cells. Therefore, ACSS2 holds promise as a potentially significant target for cancer treatment.

In non-ruminant animals, ACLY is responsible for catalyzing the conversion of citric acid into acetyl-CoA [23]. Recent investigations have indicated that knocking out the ACLY gene leads to a deceleration in cell proliferation. However, normal cellular growth is restored by introducing fatty acids and cholesterol into the cell culture medium. Simultaneously, this knockout also significantly upregulates the expression of the ACSS2 gene, accelerating the de novo synthesis of fatty acids that heavily rely on ACSS2 to provide acetyl-CoA. This observation indicates reciprocal compensation between the ACLY and ACSS2 genes [24]. In this study, siRNA-mediated interference technology was employed to interfere with ACSS2, detecting its effects on fatty acid synthesis and triglyceride deposition and verifying its ability to promote the secretion of triglycerides and cholesterol. Results reported were consistent with those reported earlier. Taking this as an entry point, we investigated the ACSS2 regulatory mechanism, providing a rationale for further clarifying fatty acid synthesis in the bovine mammary gland.

It is important to take into account the abundance of circRNA expression when studying its function, which is relative to its linear RNA copy (mRNA or lncRNA) [25]. Despite the fact that most circRNAs are not as abundant as linear RNAs, they can be found in greater abundance in certain cases as well. CircRNAs exhibit increased stability due to the absence of free ends, but they may be downregulated in certain conditions, like colorectal cancer and Alzheimer’s [26]. A closer look at how circRNA levels are affected by various conditions in different tissues is therefore necessary. circRNA localization in cells is the main focus of studies on its function. There has been evidence that exon-derived circRNAs primarily exist in the cytoplasm, but it is worth exploring whether intron-derived circRNAs and EIciRNAs are located in the nucleus or in the cytoplasm [27]. This will provide the foundation for understanding the function of circRNAs and the treatment methods based on circRNAs. In our study, circRNA-02213 was verified to promote milk fat metabolism and cell differentiation in dairy cows, as proven by experiments such as measuring triglycerides, cholesterol, and Oil Red O. In this regard, circRNA-02213 appears to be a relatively powerful circRNA.

CircRNAs and miRNAs competing for binding sites have the potential to affect the translation or stability of mRNAs. In addition, circRNAs are capable of directly interacting with other RNA molecules, such as mRNAs and lncRNAs [28]. These interactions may influence the stability, translation, and localization of both mRNAs and lncRNAs. For example, mRNAs forming a loop can improve their translation efficiency because circRNAs bring the 5′ and 3′ ends of the target mRNA closer together [29]. Furthermore, circRNAs are capable of interacting with RBPs and even function as their sponges. CircRNAs interacting with RBPs may potentially influence the splicing, transportation, storage, and translation of target mRNAs. Conversely, binding RBPs to mRNAs may modify the formation, function, abundance, or subcellular positioning of circRNAs [30].

It should be noted that, although circRNAs are thought to be stable, their turnover has remained unexplored when RBP levels interacting with them are altered. Similarly, circRNAs interacting with transcription factors (TF) can influence TF translocation to the nucleus or modulate their transcriptional activity [31]. For example, circ-Foxo3 can be used as bait for TF [32]. Based on software prediction analysis, this study has unveiled the existence of a binding site between circRNA-02213 and miR-328. Software prediction and dual luciferase reporter gene detection system analysis have substantiated that circRNA-02213 promotes milk fat metabolism by adsorbing miR-328.

## 5. Conclusions

**Figure 8:**
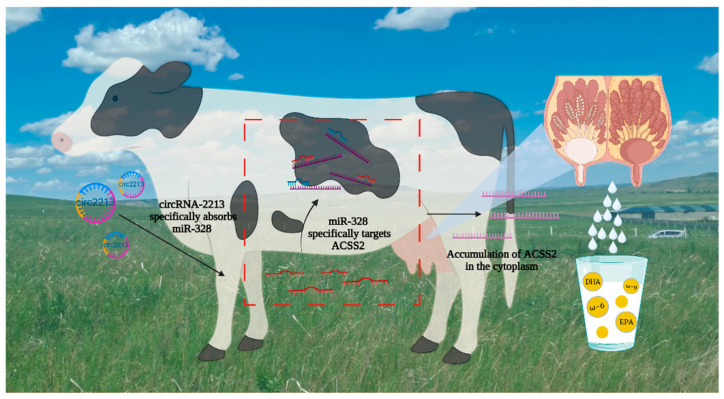
Mechanism of circRNA-02213/miR-328/ACSS2 pathway regulating milk fat metabolism in bovine mammary epithelial cells.

circRNA-02213 and miR-328 exhibit contrasting effects on the differentiation and apoptosis of myoblasts. circRNA-02213 promotes triglyceride levels—an effect that the overexpression of miR-328 can counteract. Moreover, circRNA-02213 promotes triglycerides, cholesterol, and lipid droplets, and cell function remains similar to that of the control group when circRNA-02213 and miR-328 are both overexpressed, indicating the effectiveness of circRNA-02213 as a molecular sponge for miR-328 (Figure 8).

## Data Availability

All data in this manuscript have not been published. The materials, data, and associated protocols of this study are available from the corresponding author upon request.

## Supplementary Materials

The following supporting information can be downloaded at: https://www.mdpi.com/article/10.3390/genes16111351/s1, Table S1: Vector construction primers. Table S2: Primers for genes. Table S3: The sequence of circRNA-02213. Table S4: circRNA11228 construction primers.
