# Supplementary material for: circRNA-02213 Regulates Milk Fat Synthesis in Bovine Mammary Epithelial Cells via ACSS2
Source: Genes (Basel). 2025 Nov 9;16(11):1351. doi: 10.3390/genes16111351 (PMC12652434; doi:10.3390/genes16111351)
Supplement: Supplementary file 1 [file genes-16-01351-s001.zip › genes-3964318-supplementary.pdf]

Table S1: Vector construction primers

Primers name	Primer sequence 5'-3'
Psi-CHECK-Circ RNA02213-w	CCGCTCGAGCCTGGCGCCCCAGGACAGTGGAGGGAGGCGAGGGC CAGTCCAACGGCGCGGCGTCTCGCCGGCGTAAGAATA
Psi-CHECK-Circ RNA02213-Mut	CCGCTCGAGCCTGGCGCCCCAGGACAGTTTTTTTTTTTTGGGCCA GTCCAACGGCGCGGCGTCTCGCCGGCGTAAGAATA
ACSS2-up	ccgctcgagATCCTGCCTGGAACCCAGGA
ACSS2 -Down	CTCTAATCCTATGTCATCTcgccggcgtataaa
ACSS2-overlap	GGAGUGCUGTTTTTTTGUGC

Table S2: Primers for genes

Gene	Primer sequence (5′ to 3′)	Size(bp)
DGAT1	CCACTGGGACCTGAGGTGTC	111
	GCATCACCACACACCAATTCA	
DGAT2	CATGTACACATTCTGCACCGATT	100
	TGACCTCCTGCCACCTTTCT	
GPAM	GCAGGTTTATCCAGTATGGCATT	63
	GGACTGATATCTTCCTGATCATCTTG	
PLIN2	GGGCTCCACCACCGTGTTCCA	226
	GCTCTGCTGGGCCTGCAGCTG	
AGPAT6	AAGCAAGTTGCCCATCCTCA	101
	AAACTGTGGCTCCAATTTCGA	
FASN	GGGCTCCACCACCGTGTTCCA	226
	GCTCTGCTGGGCCTGCAGCTG	
SCD1	CCATCGCCTGTGGAGTCAC	257
	GTCGGATAAATCTAGCGTAGCA	
ELOVL6	GGAAGCCTTTAGTGCTCTGGTC	205
	ATTGTATCTCCTAGTTCGGGTGC	
FABP3	GATGAGACCACGGCAGATG	120
	GTCAACTATTTCCCGCACAAG	
CD36	GTACAGATGCAGCCTCATTTCC	81
	TGGACCTGCAAATATCAGAGGA	
ATGL	GGAGCTTATCCAGGCCAATG	180
	TGCGGGCAGATGTCACTCT	
HSL	GGGAGCACTACAAACGCAACG	118
	TGAATGATCCGCTCAAACTCG	
ACOX1	CGAGTTCATTCTCAACAGTCCT	211
	GCATCTTCAAGTAGCCATTATCC	
CPT1	AAGGACCTCTACGCCAACACG	267
	TTTGCGGTGGACGATGGAG	
PPARγ	CCTTCACCACCGTTGACTTCT	145
	GATACAGGCTCCACTTTGATTGC	
ACSS2	GGCGAATGCCTCTACTGCTT	100

	GGCCAATCTTTTCTCTAATCTGCTT	
UXT	TGTGGCCCTTGGATATGGTT	101
	GGTTGTCGCTGAGCTCTGTG	

Table S3: The sequence of circRNA-02213

**Name**	**Sequence**
>circRNA_02213	aacaaAGTAATTCTGGTTTTTCAAGCCAGTAGAGGAT<br>GGATTCCCTGCAAACACGTCTCTAGCCTCACTG<br>ACCTCCTCCAGCCTTGCGCACCCTGAGCAGACCCT<br>GGTGCCCACACCCCAGCTCTTACCCTGCCCTGGAC<br>CACACTGCCTGGCGCCCCAGGACAGTGGAGGGAG<br>GCGAGGGCCAGTCCAACGGCGCGGCGTCTTCCTG<br>TC<br>CCGCACGTGGCCTGCGATGGGATGGACAAAGAGG<br>AAGAGAACCACTACGTTTCGCAACTTAGGGACGT<br>CT<br>ACAGCAGCTGTGACACCACGGGGACAGGCTTCTT<br>GGACCGGGAGGAGCTGACCCAGCTCTGCCTGAAG<br>CT<br>CCATCTGGAAAAACAGCTGCCTGTCCTCCTGCACA<br>CACTTCTCGGAAACAACCAGTTTGCCAGGGTTAAC<br>TTCGAGGAATTTAAGGACGGTTTCATAGCTGTGTT<br>GTCATCCCAGTCTGGTCTTGCCTCCTCAGATGAAG<br>ACAGTGGGTCTTTGGAGTCAG

Table S4: circRNA11228 construction primers

**Primers name**	**Primer sequence 5’-3’**
pcDNA-circRNA11228-F	CCCAAGCTTGTAATTCTGGTTTTTCAAGC
pcDNA-circRNA11228-R	GGGGTACCGAAGACAGTGGGTCTTTGGA
